# Precision prediction of intervertebral disc degeneration in ankylosing spondylitis using a nomogram model reveals the pivotal role of Th2-type immune dysregulation

**DOI:** 10.3389/fimmu.2025.1556738

**Published:** 2025-05-12

**Authors:** Xiao-nan Wang, Xiao-tian Ma, Jie Li, Run-tian Zhou, Yuan-zhang Jin, Xiao-feng Zhao, Dou-dou Jing, Bin Zhao

**Affiliations:** ^1^ Department of Orthopaedics, The Second Clinical Medical College of Shanxi Medical University, Taiyuan, China; ^2^ Academy of Medical Sciences, Shanxi Medical University, Taiyuan, China

**Keywords:** precision medicine, ankylosing spondylitis, intervertebral disc degeneration, nomogram, Th2 cells, IL-4, chronic inflammation, immune imbalance

## Abstract

**Background:**

Ankylosing spondylitis (AS) is an immune-mediated chronic inflammatory disease. When AS is complicated by intervertebral disc degeneration (IVDD), disease complexity increases substantially, often resulting in poor long-term outcomes. Although previous studies have explored the mechanisms linking AS and IVDD, reliable tools for precise risk prediction and early intervention remain scarce.

**Methods:**

In this retrospective study, we enrolled 144 patients with AS (60 with and 84 without IVDD). Their clinical features, immune status, and inflammatory cytokine levels were analyzed. A nomogram prediction model was constructed using multivariable logistic regression. Model performance was evaluated via receiver operating characteristic (ROC) curve, calibration curve, and decision curve analysis (DCA).

**Results:**

Multivariable analysis identified body mass index (BMI), peripheral blood Th2 cell percentage (Th2%), and serum IL-4 levels as independent risk factors for IVDD in patients with AS. All associations remained statistically significant after Benjamini–Hochberg correction (BMI: BH-adjusted P = 0.001; Th2%: BH-adjusted P = 0.019; IL-4: BH-adjusted P = 0.019). Incorporation of these factors into a nomogram yielded excellent discriminative performance (area under the curve, AUC = 0.83) and calibration, outperforming a simplified model based solely on BMI (AUC = 0.74). This improvement in predictive accuracy was statistically significant, as determined by DeLong’s test (P = 0.018). DCA revealed that at a threshold probability of 60.8%, the nomogram effectively distinguished high-risk patients, underscoring its strong clinical applicability.

**Conclusions:**

This study is the first to highlight the critical roles of Th2 cells and IL-4 in AS complicated by IVDD, and establishes a nomogram that accurately predicts the risk of IVDD in AS. Beyond offering a tool for early detection and personalized management, these findings open avenues for investigating overlapping pathogenic mechanisms and potential immunotherapeutic targets in AS-IVDD.

## Introduction

Ankylosing spondylitis (AS) is a prevalent immune-mediated chronic inflammatory disease, affecting primarily young adult males, with a global prevalence of approximately 0.1%–1.4% and 0.3%–0.5% in Asian populations (1, 2). The hallmark pathological changes in AS include loss of normal spinal curvature, excessive bone formation, and fibrosis, leading to a marked decline in patients’ quality of life (3). Intervertebral disc degeneration (IVDD) is a common complication of AS, exacerbating pain, functional impairment, and neurological deficits, while also worsening long-term prognosis, posing a major challenge in AS management (4, 5).

Compared with individuals who develop IVDD in the general population, those with AS and IVDD present more complex pathological and clinical manifestations (6). The interplay between chronic inflammation and aberrant ossification accelerates disc matrix senescence and worsens spinal biomechanics, compounding the therapeutic challenge (7, 8). Conventional non-surgical treatment often shows limited efficacy in patients with AS and IVDD. Surgical intervention, on the other hand, is high-risk and complex due to the spinal rigidity and proliferative changes characteristic of AS, resulting in increased difficulty during procedures such as screw placement and decompression (9). Concurrently, a persistent state of immune hyperactivation in AS raises the likelihood of complications such as intraoperative bleeding, postoperative infection, impaired wound healing, and venous thromboembolism (10). These risks lead to significant variability in surgical outcomes, with some cases failing to achieve the desired therapeutic benefit (11). Consequently, early identification of high-risk patients and individualized treatment has become paramount.

At present, clinical diagnosis and risk prediction for AS-IVDD primarily rely on imaging examinations and disease course observations (12). Yet conventional imaging methods tend to focus on structural alterations, lacking sensitivity to molecular and cellular changes in the disease’s early stages (13). Moreover, due to significant abnormalities in the spinal anatomy and biomechanical properties of AS patients, the manifestations of IVDD can exhibit marked heterogeneity, rendering traditional diagnostic criteria inadequate in sensitivity and specificity (14). These limitations may cause delayed recognition, misdiagnosis, or missed optimal therapeutic opportunities. Thus, there is an urgent need for an approach that can capture disease-related pathophysiological processes at both molecular and cellular levels early on, to optimize patient management and improve outcomes.

Nomogram modeling, a pivotal component of precision medicine, integrates multivariable data to yield individualized risk predictions (15). By combining metabolic, immunological, and inflammatory parameters, nomograms can tailor prognostic estimates and enable early identification of high-risk populations (16, 17). However, in the context of AS complicated by IVDD, existing models often limit their scope to single clinical or imaging parameters, lacking comprehensive data integration and robust clinical validation (18, 19). Consequently, they fail to adequately characterize the intricate pathophysiology of this disorder or identify putative therapeutic targets.

Against this backdrop, our study aimed to systematically investigate the clinical characteristics and immune features of AS-IVDD, and to build and validate a multi-omics-based nomogram for precise identification of individuals at high risk. By incorporating metabolic indices, immune-cell profiles, and inflammatory cytokine levels, the proposed model seeks to provide a valuable clinical decision-making tool and shed new light on the overlapping pathophysiological mechanisms of AS and IVDD. Our ultimate goal is to advance the implementation of precision medicine in managing AS complicated by IVDD, enhance long-term patient outcomes, and facilitate the development of innovative therapeutic strategies.

## Methods

This retrospective study included 144 AS patients (110 males and 34 females) admitted to the Department of Rheumatology or Orthopedics at the Second Hospital of Shanxi Medical University between June 2022 and June 2024. Inclusion criteria were based on either the 1984 revised New York criteria (20) and/or the 2009 ASAS classification criteria (Assessment of SpondyloArthritis International Society) (21, 22). Patients with other autoimmune diseases, active infections, malignancies, or pregnancy were excluded. Comprehensive clinical, laboratory, and radiological data were gathered, including complete blood count, serum lipids, C-reactive protein (CRP), erythrocyte sedimentation rate (ESR), liver enzymes, bilirubin, immunoglobulins, peripheral blood lymphocyte subpopulations, and cytokine levels. We confirm that the dataset was carefully screened prior to analysis, and only patients with complete clinical, laboratory, and flow cytometry data were included. As a result, no imputation was required, and there were no missing data in the final analytical cohort. All patients underwent lumbar MRI examination, evaluated by Pfirrmann grading; those graded >III with classic clinical manifestations were diagnosed with IVDD (23). Participants were then assigned to either the AS group (n = 84) or the AS-IVDD group (n = 60). Ethical approval was granted by the institutional review board of The Second Hospital of Shanxi Medical University [Approval No. 2024YX (369)].

### Flow cytometry for peripheral blood lymphocyte subsets

Heparinized venous blood was collected from each participant. Peripheral blood mononuclear cells (PBMCs) were isolated by Ficoll-Hypaque density-gradient centrifugation, adjusted to appropriate cell concentrations, and labeled with fluorochrome-conjugated monoclonal antibodies. T lymphocytes were stained with anti-CD3-FITC, anti-CD8-PE, anti-CD45-PerCP, and anti-CD4-APC; B and NK cells with anti-CD3-FITC, anti-CD16+CD56-PE, anti-CD45-PerCP, and anti-CD19-APC; Th1, Th2, and Th17 cells with anti-CD4-FITC/anti-IFN-γ-APC, anti-CD4-FITC/anti-IL-4-PE, and anti-CD4-FITC/anti-IL-17A-PE, respectively; and Tregs with anti-CD4-FITC, anti-CD25-APC, and anti-FOXP3-PE. Antibodies were purchased from BD Biosciences (Franklin Lakes, NJ, USA) and used according to manufacturer protocols. Absolute and relative counts of lymphocyte subsets were determined using a FACSCalibur™ flow cytometer and BD Multitest™ software (BD Biosciences).

### Flow cytometry for cytokine levels

Serum was separated by centrifugation within 1 h of blood collection and stored at −20°C. A flow cytometry-based assay was employed to detect seven cytokines—IL-2, IL-4, IL-6, IL-10, IL-17, IFN-γ, and TNF-α—using CBA kits (Cellgene Biotech, Jiangxi, China). All procedures followed the manufacturer’s instructions to ensure accuracy and reliability.

### Statistical analysis

All statistical analyses were performed using R software (version 4.3.3). For continuous variables, normality was assessed using the Shapiro–Wilk test. Variables following a normal distribution were presented as mean ± standard deviation (SD) and compared using the independent samples t-test. Non-normally distributed variables were expressed as median and interquartile range [M (P25–P75)] and analyzed using the Mann–Whitney U test. Categorical variables were reported as frequencies and percentages [n (%)] and compared using either the chi-squared test or Fisher’s exact test, as appropriate. Correlation analyses were conducted using Pearson or Spearman methods depending on variable distributions.

To control the risk of type I errors due to multiple comparisons, the Benjamini–Hochberg (BH) method was applied to adjust p-values and control the false discovery rate (FDR). Statistical significance was defined as a BH-adjusted p < 0.05. In the univariate logistic regression analysis, variables with a BH-adjusted P-value < 0.10 were retained for multivariable modeling to reduce the risk of type II error and avoid overlooking potentially important predictors of IVDD in patients with AS. To avoid multicollinearity among predictors, variance inflation factor (VIF) diagnostics were used, and variables with high collinearity were excluded. Final results were expressed as odds ratios (ORs) with 95% confidence intervals (CIs), and statistical significance was set at BH-adjusted two-sided p < 0.05.

A nomogram prediction model was constructed based on variables that remained statistically significant in the multivariate logistic regression analysis. Model performance was evaluated through several metrics (1): discriminatory ability was assessed using the area under the receiver operating characteristic curve (AUC) and the concordance index (C-index); bootstrap resampling (1,000 iterations) was employed to obtain bias-corrected AUC estimates, reflecting the model’s robustness and predictive accuracy (2); model calibration was examined by plotting calibration curves generated through bootstrap resampling and performing the Hosmer–Lemeshow goodness-of-fit test (3); clinical utility and net benefit were assessed using decision curve analysis (DCA) and clinical impact curves (CIC); and (4) the DeLong test was used to compare the AUCs of alternative models, with p < 0.05 indicating a statistically significant difference. Data visualization was performed using R (version 4.3.3).

## Results

### Comparison of demographic, clinical, and laboratory variables

A total of 144 AS patients were enrolled, including 60 with IVDD (AS-IVDD group) and 84 without (AS group). Baseline demographics (sex, age, smoking, alcohol use), disease duration, and treatments (NSAIDs, glucocorticoids, conventional synthetic DMARDs, and biologic DMARDs) were matched between groups (**Table 1**). Although the AS-IVDD group showed a slightly longer disease duration (median 32.50 vs. 30.50 months), this difference was not statistically significant (BH-adjusted P = 0.821). Notably, BMI was significantly higher in the AS-IVDD group compared with the AS group [25.51 (23.41, 29.32) vs. 23.34 (20.57, 24.92), BH-adjusted P < 0.001], suggesting that BMI may be a potential risk factor for IVDD among AS patients. Additionally, the AS-IVDD group had higher monocyte counts [0.47 (0.36, 0.68) vs. 0.39 (0.30, 0.52), unadjusted P = 0.005]. However, this difference did not remain statistically significant after Benjamini–Hochberg adjustment (BH-adjusted P = 0.066). In terms of organ function and lipid metabolism, no significant differences were found. However, CRP levels were significantly elevated in the AS-IVDD group [6.92 (2.36, 16.33) vs. 3.20 (1.45, 6.25), BH-adjusted P<0.001], reflecting a higher inflammatory burden among these patients. No significant differences were noted in ESR or levels of immunoglobulins (IgA, IgG, IgM).

**Table 1:** Clinical characteristics of AS-IVDD group and AS group.

Characteristics	AS-IVDD (n = 60)	AS (n = 84)	t/z/χ^2^	Unadjusted P-value	BH-adjusted P value
Demographics					
Female (%)	13 (21.67)	21 (25.00)	0.216	0.642	0.841
Male (%)	47 (78.33)	63 (75.00)			
Age (years)	48.00 (42.75, 54.50)	48.50 (36.00, 55.00)	0.594	0.552	0.821
BMI (Kg/m^2^)	25.51 (23.41, 29.32)	23.34 (20.57, 24.92)	5.015	<0.001^***^	<0.001^***^
Course of disease (month)	32.50 (24.00, 38.00)	30.50 (21.75, 42.50)	0.741	0.459	0.821
Smoke (%)	10 (16.67)	13 (15.48)	0.037	0.848	0.946
Drink (%)	9 (15.00)	12 (14.29)	0.014	0.905	0.967
Hypertension (%)	12 (20.00)	11 (13.10)	1.243	0.265	0.821
Diabetes (%)	11 (18.33)	16 (19.05)	-0.012	0.914	0.967
Treatment					
NASIDs (%)	35 (58.33)	45 (53.57)	0.321	0.571	0.821
CS (%)	10 (16.67)	17 (20.24)	-0.293	0.588	0.821
CsDMARDs (%)	13 (21.67)	14 (16.67)	0.574	0.449	0.821
bDMARDs (%)	5 (8.33)	4 (4.76)	0.762	0.383	0.821
Laboratory Characteristics					
WBC (*10^9^/L)	6.09 (5.07, 7.24)	6.22 (5.38, 7.95)	-0.810	0.418	0.821
RBC (*10^12^/L)	4.39 (4.12, 4.89)	4.61 (4.17, 4.87)	-0.962	0.336	0.821
HB (g/L)	135.00 (122.00, 148.50)	134.00 (123.75, 148.00)	0.176	0.860	0.946
PLT (*10^9^/L)	262.50 (214.00, 294.25)	270.50 (229.75, 313.00)	-1.398	0.162	0.821
NE (*10^9^/L)	3.62 (2.89, 5.17)	4.13 (3.23, 5.20)	-0.928	0.353	0.821
LY (*10^9^/L)	1.76 (1.41, 2.16)	1.78 (1.45, 2.20)	-0.798	0.425	0.821
MONO (*10^9^/L)	0.47 (0.36, 0.68)	0.39 (0.30, 0.52)	2.813	0.005^**^	0.066
ALT (U/L)	18.55 (13.00, 30.20)	18.20 (13.78, 25.07)	0.646	0.518	0.821
AST (U/L)	21.20 (16.83, 25.30)	19.90 (16.25, 24.18)	0.588	0.557	0.821
TBIL (μmol/L)	11.11 (8.98, 13.55)	11.85 (9.08, 14.63)	-0.657	0.511	0.821
DBIL (μmol/L)	2.05 (1.50, 2.60)	1.80 (1.60, 2.60)	0.016	0.987	0.995
IBIL (μmol/L)	9.20 (7.00, 11.03)	9.50 (7.28, 12.23)	-0.729	0.466	0.821
Cr (μmol/L)	62.25 (52.75, 73.85)	60.55 (51.00, 72.28)	0.383	0.702	0.874
CHOL (mmol/L)	4.61 (3.92, 5.10)	4.39 (4.04, 5.19)	0.217	0.828	0.946
TG (mmol/L)	1.47 (1.08, 1.96)	1.35 (0.96, 2.02)	0.436	0.663	0.842
HDL (mmol/L)	1.17 (0.98, 1.48)	1.29 (1.03, 1.46)	-0.812	0.417	0.821
LDL (mmol/L)	2.42 (2.12, 2.87)	2.60 (2.05, 3.19)	-1.031	0.303	0.821
ESR (mm/h)	13.00 (8.00, 29.25)	11.00 (7.00, 22.75)	1.002	0.316	0.821
CRP (mg/ml)	6.92 (2.36, 16.33)	3.20 (1.45, 6.25)	3.828	<0.001^***^	<0.001^***^
IgA (g/L)	2.12 (1.59 3.13)	2.27 (1.78 3.05)	-0.231	0.817	0.946
IgG (g/L)	10.32 (8.56, 13.29)	10.30 (8.85, 12.01)	0.006	0.995	0.995
IgM (g/L)	0.95 (0.61, 1.32)	0.92 (0.63, 1.29)	0.612	0.541	0.821

BMI, body mass index; NSAID, nonsteroidal anti-inflammatory drug; CS, Conventional synthetic; CsDMARDs, Conventional synthetic disease-modifying antirheumatic drugs; bDMARDs, Biological disease-modifying antirheumatic drugs; WBC, white blood cells; RBC, Red blood cells; Hb, Hemoglobin; PLT, platelets; NE, Neutrophilic granulocyte; LY, lymphocytes; MONO, Monocyte; ALT, Alanine transaminase; AST, Aspartic transaminase; TBIL, Total bilirubin; DBIL, Direct bilirubin; IBIL, Indirect bilirubin; Cr, Creatinine; CHOL, Cholesterol; TG, Triglycerides; HDL, High-density lipoprotein; LDL, Low-density lipoprotein; ESR, erythrocyte sedimentation rate; CRP, C-reactive protein; IgA, Immunoglobulin A; IgG, Immunoglobulin G; IgM, Immunoglobulin M.

**p < 0.01, ***p < 0.001.

### Elevated peripheral Th2 cells and decreased Tregs in AS-IVDD

**Figure 1:**
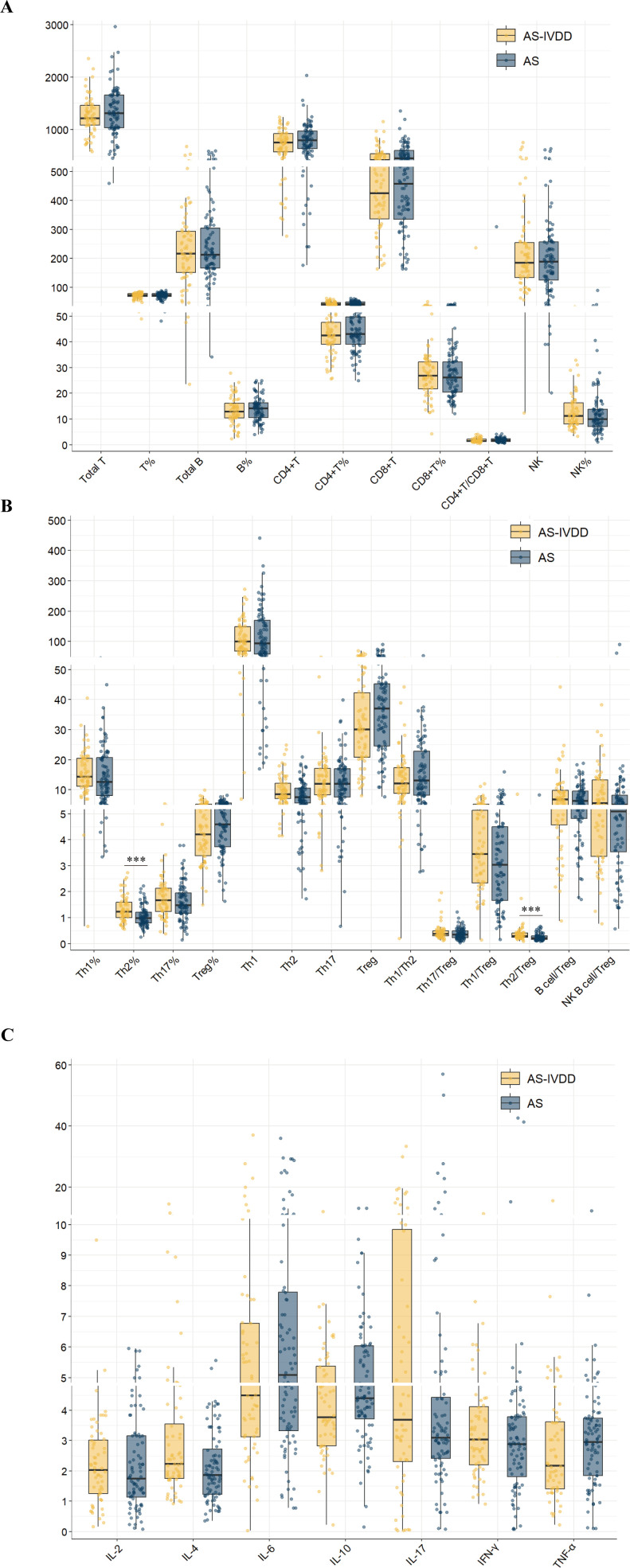
Peripheral blood lymphocyte subpopulation levels in both groups. **(A)** Comparison of lymphocyte subsets in peripheral blood between two groups. **(B)** Comparison of levels of functional CD4 + T cells in peripheral blood between two groups. **(C)** Comparison of cytokine levels between the two groups. (***p < 0.001).

To dissect immune status differences, peripheral lymphocyte subsets were examined (**Supplementary Table S1**). Th2 cell percentage (Th2%) was significantly higher in the AS-IVDD group than in the AS group (BH-adjusted P<0.001). The Th2/Treg ratio also markedly increased (BH-adjusted P < 0.001), indicating a pronounced Th2-dominant immune imbalance (**Figure 1B**). Concomitantly, absolute Treg counts were lower in the AS–IVDD group (unadjusted P = 0.046); however, this difference did not remain statistically significant after correction for multiple comparisons (BH-adjusted P = 0.380). While not conclusive, this trend may point toward a potential impairment in Treg-mediated immunoregulation in AS-related disc degeneration, warranting further validation in larger, independent cohorts. By contrast, other subpopulations, including total T cells, B cells, CD4^+^ T cells, CD8^+^ T cells, and related ratios, did not differ significantly between the two groups (**Figure 1A**).

### Elevated IL-4 levels in AS-IVDD

To preliminarily assess potential immune-related risk factors for AS–IVDD, we compared serum cytokine profiles between groups (**Supplementary Table S2**). Patients with AS–IVDD exhibited higher IL-4 levels than those with AS alone (unadjusted P = 0.008; **Figure 1C**), although this difference did not remain statistically significant after Benjamini–Hochberg adjustment (BH-adjusted P = 0.088). No significant differences were observed for other cytokines (IL-2, IL-6, IL-10, IL-17, IFN-γ and TNF-α; **Supplementary Table S2**). These results suggest a trend toward elevated IL-4 in the AS–IVDD group, meriting further investigation. Collectively, these findings point to a potential involvement of Th2-skewed immune responses in AS–IVDD pathogenesis and underscore the need for more rigorous statistical analyses to confirm these associations.

### Construction of the nomogram for predicting AS-IVDD

**Figure 2:**
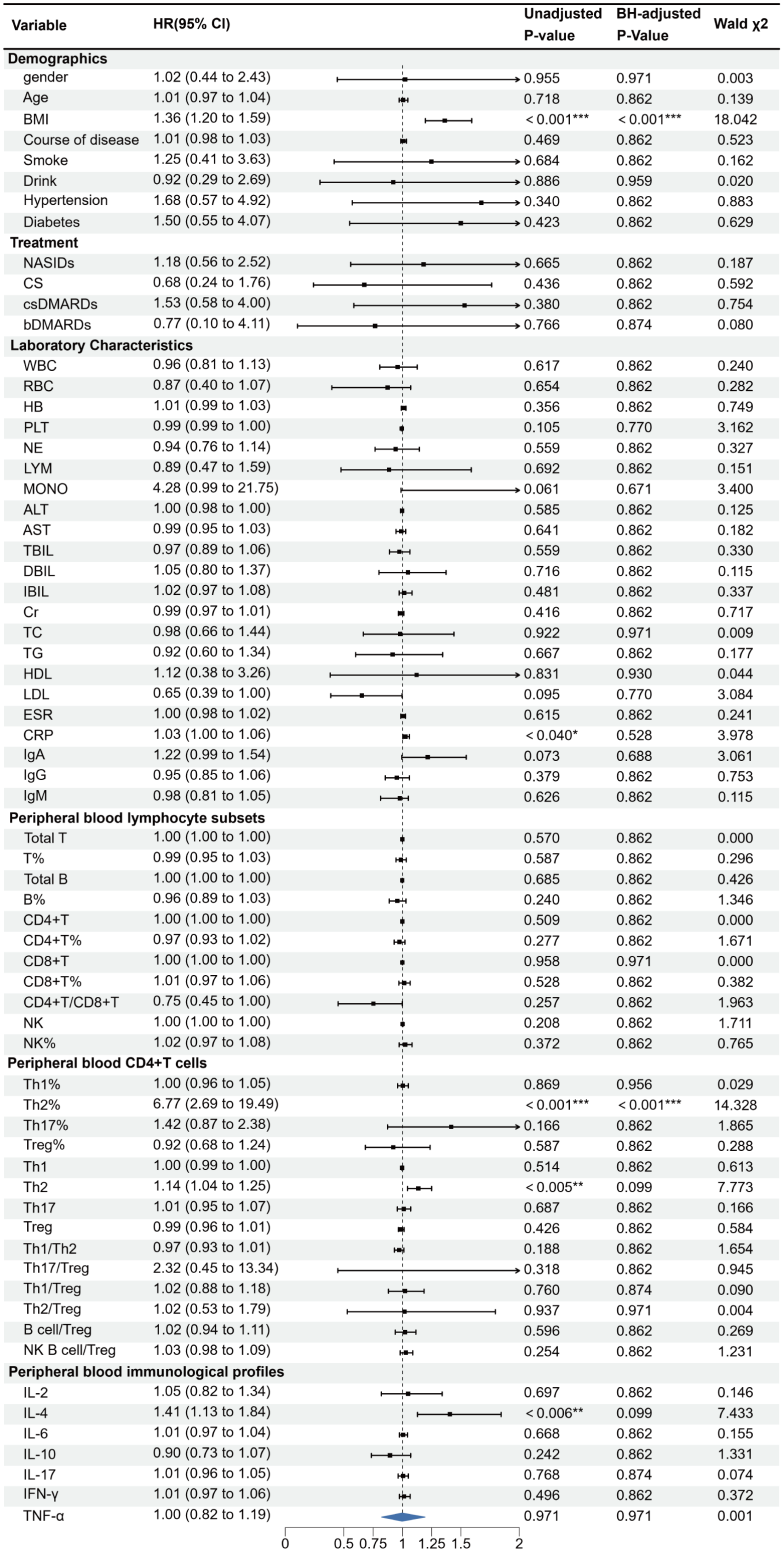
Univariate logistic regression analyses for factors associated with the presence of IVDD in AS patients. (*p < 0.05, **p < 0.01, ***p < 0.001). OR, odds ratio; 95%CI:95% confidence interval.

**Figure 3:**
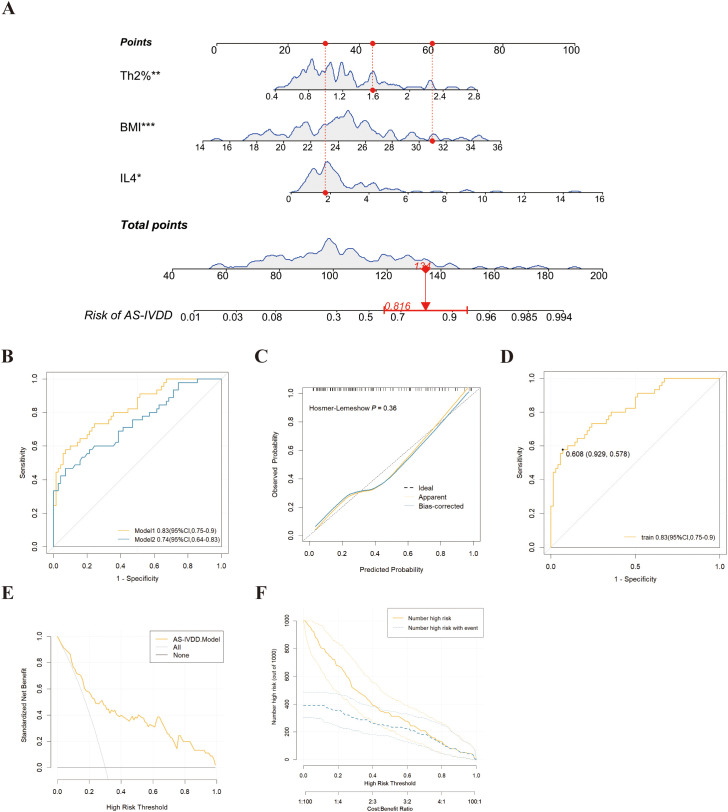
Development and assessment of a nomogram for prediction of IVDD in AS. **(A)** Example of prediction nomogram for risk of IVDD in AS patients. The nomogram incorporates the risk factors of Th2%, IL-4 and BMI. **(B)** The receiver operating characteristic (ROC) curve for the discrimination of the nomogram to predict the risk of IVDD in AS patients. Model 1: The nomogram incorporating the risk factors of Th2%, IL-4 and BMI; Model 2: The nomogram incorporating the risk factors of BMI. **(C)** Calibration curve for predicting the risk of IVDD in AS patients in training cohorts. **(D)** The ROC curve for the discrimination of the nomogram to predict the risk of IVDD in training cohorts. **(E)** Decision curve analysis for predicting the risk of IVDD in AS patients in training cohorts. **(F)** Clinical Impact Curve (CIC) response prediction model for clinical effectiveness in training cohorts. *p<0.05, **p<0.01, ***p < 0.001.

All 144 AS patients were randomized (8:2 ratio) into a training set (n = 115) and a validation set (n = 29). No statistical differences were observed between the two sets, confirming their comparability. Univariate logistic regression identified BMI, Th2%, Th2 cell count, and IL-4 as potential risk factors for AS-IVDD (**Figure 2**). Evaluation of variance inflation factors (VIF < 10) ruled out significant multicollinearity among these variables (BMI = 1.0276, Th2% = 1.6069, Th2 cell count = 1.6127, and IL-4 = 1.0259). Subsequent multivariable logistic regression identified BMI, peripheral Th2%, and serum IL-4 levels as independent predictors of AS–IVDD, with Benjamini–Hochberg–adjusted P-values of 0.001, 0.019, and 0.019, respectively (**Supplementary Table S3**). Incorporating these three predictors, we developed a nomogram for personalized AS-IVDD risk estimation (**Figure 3A**). Recognizing that Th2% and IL-4 are not traditional risk factors for IVDD, we also generated a simplified model containing only BMI and evaluated both models’ performance.

### Validation of the nomogram

Nomogram Model 1 incorporated BMI, Th2%, and IL-4, while Model 2 included BMI alone. As shown in **Figure 3B** and **Table 2**, Model 1 achieved an AUC of 0.83 (95% CI: 0.68–0.85) and an optimism-corrected C-index of 0.81, compared to an AUC of 0.74 (95% CI: 0.55–0.76) and a C-index of 0.74 for Model 2. Comparative analysis using the DeLong test confirmed that Model 1 significantly outperformed Model 2 in discriminative ability (P = 0.018), supporting the added predictive value of immune biomarkers in the model. For Model 1, the AUC values in both the training and validation cohorts were 0.83 (95% CI: 0.75–0.90) and 0.81 (95% CI: 0.63–0.99), respectively (**Supplementary Figure S1A**), demonstrating robust discriminative power. To enhance confidence in the predictive performance of our model, we performed internal validation using 1,000-fold bootstrap resampling. The bias-corrected AUC was 0.81 (95% CI: 0.78–0.82), demonstrating that the model retains good discriminatory ability and robustness against overfitting. Calibration curves based on 1,000 bootstrap resamples demonstrated excellent concordance between predicted probabilities and observed outcomes (**Figure 3C**). A Hosmer–Lemeshow test (P = 0.36) further supported good model fit.

**Table 2:** Multivariate logistic regression analyses for factors associated with the presence of IVDD in AS patients.

Variables	Model 1		Model 2		P-value
	OR (95% CI)	P-value	OR (95% CI)	P-value	
BMI	1.31 (1.14-1.54)	0.001^**^	1.24 (1.10-1.42)	0.001^**^	0.018^*^
Th2%	5.25 (1.80-17.34)	0.004^**^			
IL4	1.47 (1.11-2.03)	0.012^*^			
AUC	0.83 (95%CI: 0.75-0.90)		0.74 (95% CI: 0.64-0.83)		
C-Index	0.81		0.74		

*p<0.05, **p<0.01, ***p < 0.001.

To evaluate the clinical utility of our model, we performed DCA. The results demonstrated that, across a threshold probability range of 0.1 to 1.0, using the model to guide intervention yielded a greater net clinical benefit compared to strategies of treating all or no patients. Notably, the optimal cutoff value identified by ROC analysis (60.8%) fell within this clinically useful threshold range (**Figure 3D**). When using 60.8% as the decision threshold for diagnosing AS-related IVDD, the model would allow approximately 16 out of every 100 at-risk AS patients to benefit from appropriate intervention without unnecessarily treating those without IVDD risk (**Figure 3E**). CIC corroborated these findings by showing that when the threshold probability exceeded 60%, the number of individuals predicted at high risk closely matched the actual incidence of AS-IVDD (**Figure 3F**). Collectively, Model 1 not only demonstrates remarkable predictive accuracy but also holds substantial promise for clinical application.

## Discussion

IVDD is a chief contributor to the global increase in disability-adjusted life years (DALYs), resulting in chronic pain and functional decline, with a substantial impact on healthcare expenditures (24, 25). In AS patients, IVDD is notably more prevalent, likely due to immune dysregulation and mechanical destabilization. Moreover, IVDD in AS often has a rapid course and poses enormous therapeutic challenges (26). Advanced surgical procedures typically face heightened difficulty in AS-IVDD (27, 28) because of (1): rigid spines and local ossification complicating exposure and decompression (2); higher rates of implant failure and poor fusion outcomes (3); chronic inflammation and persistent immune activation, which raise the likelihood of infection and thrombosis. Hence, surgery is often not the first choice in AS-IVDD. Rather, early identification of high-risk individuals and refinement of interventions are crucial to mitigating the disease burden and surgical risks.

Through univariate and multivariate analyses, our findings reveal that Th2% and IL-4 are independent risk factors for IVDD in AS, highlighting a central role of Th2-associated immune dysregulation. Our data add to prior evidence implicating Th2 responses in AS pathophysiology, suggesting that an elevated Th2 response might foster disc degeneration by diverse molecular pathways (29, 30). We integrated these immunological parameters with the more traditional risk factor BMI (31) to construct a novel IVDD risk nomogram for AS patients. Without Th2% and IL-4, the model’s predictive performance (AUC) dropped from 0.83 to 0.74, underscoring these immunologic variables as key contributors. DCA and CIC confirmed the model’s robust clinical utility, indicating that when the decision threshold is set at 60.8%, the predicted high-risk population aligns well with real-world IVDD incidence in AS, thus guiding targeted interventions. To further evaluate whether the observed performance gain justifies the added complexity and cost associated with incorporating immunological biomarkers, we conducted a DCA directly comparing the two models. As shown in **Supplementary Figure S1C**, Model 1 consistently yielded a higher net clinical benefit than Model 2 across a broad range of threshold probabilities (10%–100%). These findings suggest that, in real-world clinical settings—particularly for patients at intermediate risk—Model 1 may offer superior utility by enabling more precise risk stratification and supporting earlier, more targeted intervention strategies.

Through comprehensive multi-omics analyses, this study is the first to systematically elucidate how elevated Th2 responses and IL-4 contribute to disc pathology in the context of AS. Th2 cells typically secrete IL-4, IL-5, and IL-13, which are often associated with anti-inflammatory and reparative roles (32, 33). However, in AS-IVDD, heightened Th2 activity and excess IL-4 may paradoxically drive disc deterioration through several putative mechanisms (34, 35) (1): activation of matrix metalloproteinases (MMP-3, MMP-13), degrading disc extracellular matrix (ECM) (2); upregulation of chemokines that recruit inflammatory cells into the disc microenvironment (3); direct inhibitory effects on nucleus pulposus cell proliferation and repair. Thus, IL-4 can act as a “double-edged sword,” simultaneously regulating immune responses while undermining the integrity of the intervertebral disc. IL-4 was found to exert pleiotropic effects on intervertebral disc cells *in vitro*. Upon IL-4 stimulation, nucleus pulposus (NP) cells exhibited a pronounced catabolic phenotype, marked by elevated secretion of ECM–degrading enzymes such as MMPs and ADAMTS. Notably, IL-4 also triggered a substantial upregulation of the pro-inflammatory cytokine IL-17F, implicating a previously unrecognized role for IL-4 in reinforcing inflammatory circuits that may potentiate disc degeneration under pathological conditions (36). Previous studies have demonstrated that Th17 and Tregs contribute to the pathogenesis of chronic low back pain through immune-mediated mechanisms (37). An imbalance characterized by reduced pro-inflammatory Th17 cells and increased anti-inflammatory Tregs has been observed in the peripheral blood of affected individuals, underscoring the role of Th17/Treg crosstalk in pain and immune regulation. In contrast, our findings highlight a distinct immunological axis, wherein Th2/IL-4–driven dysregulation appears to play a more pivotal role in AS-IVDD. This may reflect the unique immune microenvironment in AS patients, the precise cellular and molecular underpinnings of which remain largely unexplored. Future studies will aim to delineate the upstream regulators and downstream effectors of the Th2/IL-4 pathway in the context of disc degeneration, with the goal of identifying potential therapeutic targets.

Our study also corroborates the longstanding observation that higher BMI is associated with an increased risk of IVDD (31, 38). Besides augmenting mechanical stress, obesity often correlates with chronic, low-grade inflammation, which heightens the activity of proinflammatory cytokines (e.g., TNF-α, IL-6), exacerbating ECM breakdown and reduced proteoglycan/collagen synthesis in the disc (39). Previous studies have shown that IL-4 is involved in mechanotransduction and catabolic responses of annulus fibrosus cells in non-degenerate intervertebral discs subjected to cyclic tensile strain (40). Although our analysis did not reveal a significant correlation between BMI, serum IL-4 levels, and the proportion of Th2 cells (**Supplementary Figure S1D**), it is plausible that these factors may act in concert to promote IVDD. The potential synergistic effects among metabolic, mechanical, and immune pathways warrant further investigation.

Strengths of this study include (1): Novel focus on Th2-mediated immunopathology. We comprehensively explored the roles of Th2% and IL-4 in the pathogenesis of AS-IVDD, broadening the current understanding of overlapping inflammatory pathways (2). Rigorous evaluation of model performance. Discrimination, calibration, and DCA collectively demonstrated that the proposed nomogram offers robust predictive value and clinical utility (3). Practical, user-friendly risk prediction. By presenting our findings through a nomogram, we provide a straightforward tool for individualized risk assessment, aligning well with the goals of precision medicine in clinical settings.

### Limitations include

(1) Owing to the relatively limited sample size, the study may have lacked sufficient power to detect subtle intergroup differences, which could impact the robustness of the statistical analyses (2). The predictive model was internally validated via bootstrap resampling, but no external test set was available due to the single-center, retrospective design. This limitation may introduce selection bias and restrict the generalizability of our findings. Future studies using large, multicenter prospective cohorts will be essential to externally validate and refine our nomogram model (3). While Th2 cells and IL-4 emerge as independent risk factors, their precise molecular mechanisms in AS-IVDD remain incompletely understood. Further investigations are needed to delineate how the Th2/IL-4 axis modulates the disc microenvironment, including its interactions with extracellular matrix turnover, inflammatory mediators, and resident disc cells. Incorporating multi-omics profiling and additional immunological biomarkers may further enhance model performance and broaden its clinical applicability.

## Conclusions

This study provides the first demonstration of a clear immune link between AS and IVDD, underscoring the pivotal roles of elevated Th2% and IL-4 levels. We show that incorporating these immune markers alongside BMI significantly improves the accuracy of a nomogram designed to predict IVDD in AS. The model’s high predictive power and strong clinical utility underscore its potential for guiding individual risk assessment, enabling earlier and more targeted interventions to mitigate disease severity and surgical risk. Moreover, our data point to immunomodulatory strategies as promising therapeutic avenues for AS-IVDD. For example, monoclonal antibodies such as dupilumab, which blocks IL-4 receptor α signaling, have already been approved for the treatment of Th2-mediated diseases and may be repurposed for inflammatory spinal disorders. In addition, agents that inhibit Th2 cell differentiation or modulate cytokine profiles may hold therapeutic promise in mitigating disc degeneration. Future investigations should include larger multicenter cohorts and functional assays to fully elucidate the underlying molecular mechanisms, thereby accelerating the development of novel treatments. Ultimately, our work signifies an important step toward precision medicine for patients with AS and IVDD, aiming to reduce disease burden and enhance long-term outcomes.

## Data Availability

The original contributions presented in the study are included in the article/**Supplementary Material**. Further inquiries can be directed to the corresponding authors.

## Supplementary material

Supplementary Figure 1
**(A)** The ROC curve for the discrimination of the nomogram to predict the risk of IVDD in training and validation cohorts. **(B)** DCA for predicting the risk of IVDD in AS patients in validation cohorts. **(C)** DCA demonstrated that Model 1 consistently provided greater net clinical benefit than Model 2 across a threshold probability range of 10% to 100%, supporting its superior utility in guiding clinical decision-making. **(D)** Spearman correlation analysis was performed to assess the relationships between BMI and immune parameters IL-4 and Th2%. The color gradient represents the strength and direction of correlation (ρ), with red indicating positive and blue indicating negative associations. A weak negative correlation was observed between BMI and IL-4 (ρ = -0.13, p = 0.22), while a weak positive correlation was found between BMI and Th2% (ρ = 0.12, p = 0.27). However, neither reached statistical significance, suggesting no meaningful correlation between BMI and these immune markers in the current dataset.
