# Supplementary material for: Precision prediction of intervertebral disc degeneration in ankylosing spondylitis using a nomogram model reveals the pivotal role of Th2-type immune dysregulation
Source: Front Immunol. 2025 May 12;16:1556738. doi: 10.3389/fimmu.2025.1556738 (PMC12104166; doi:10.3389/fimmu.2025.1556738)
Supplement: Supplementary file 1 [file Table1.doc]

TABLE S1 Absolute counts and proportions of lymphocytes in the peripheral blood in the study participants.

| **(A)** | **AS+IVDD (n = 60)** | **AS (n = 84)** | **t / z / χ^2^** | **unadjusted P-value** | **BH-adjusted**  **P value** |
| --- | --- | --- | --- | --- | --- |
| Total T (cells/mL) | 1219.31(1081.99, 1462.86) | 1316.79(1036.66, 1659.18) | -1.157 | 0.247 | 0.821 |
| T% | 73.56(68.17, 78.14) | 74.19(68.83, 78.35) | -0.470 | 0.638 | 0.841 |
| Total B (cells/mL) | 217.35(151.18, 293.96) | 213.41(165.91 304.61) | -0.209 | 0.835 | 0.946 |
| B% | 13.29±5.48 | 14.06±4.84 | -0.889 | 0.376 | 0.821 |
| CD4^+^T (cells/mL) | 755.96（576.34, 922.34） | 796.28（635.40, 974.99） | -0.983 | 0.326 | 0.821 |
| CD4^+^T% | 42.81±8.36 | 44.08± 7.78 | -0.937 | 0.351 | 0.821 |
| CD8^+^T (cells/mL) | 424.76（335.94, 539.07） | 457.68（334.78, 602.17） | -0.539 | 0.590 | 0.821 |
| CD8^+^T% | 26.50±8.14 | 26.56±7.70 | 0.041 | 0.967 | 0.995 |
| CD4^+^T/CD8^+^T | 1.53(1.25, 2.18) | 1.74(1.22, 2.35) | -0.707 | 0.479 | 0.821 |
| NK (cells/mL) | 185.18(133.81, 254.70) | 189.32(124.59, 257.07) | 0.454 | 0.650 | 0.841 |
| NK% | 11.33 (8.09, 16.28) | 10.03(7.04, 13.77) | 1.023 | 0.306 | 0.821 |
| **(B)** | **AS+IVDD (n = 60)** | **AS ( n = 84)** | **t / z / χ^2^** | **unadjusted P-value** | **BH-adjusted**  **P value** |
| Th1 (cells/mL) | 100.12(68.58, 148.21) | 94.66(58.07, 169.80) | 0.097 | 0.923 | 0.967 |
| Th1% | 14.43(11.08, 20.39) | 12.67(8.10, 20.65) | 1.193 | 0.233 | 0.821 |
| Th2 (cells/mL) | 8.58(7.02, 12.17) | 7.52(5.53, 10.49) | 2.249 | 0.025^*^ | 0.236 |
| Th2% | 1.24(1.01, 1.59) | 0.98(0.79, 1.20) | 3.893 | ＜0.001^***^ | ＜0.001^***^ |
| Th17 (cells/mL) | 12.00(8.23, 17.07) | 12.02(7.48, 16.91) | 0.245 | 0.806 | 0.946 |
| Th17% | 1.68(1.23 2.13) | 1.48(1.16, 1.95) | 1.262 | 0.207 | 0.821 |
| Treg (cells/mL) | 30.11(20.80, 42.30) | 37.07 (24.55, 45.31) | -1.994 | 0.046^*^ | 0.380 |
| Treg% | 4.21(3.38, 5.20) | 4.59(3.73, 5.58) | -1.548 | 0.122 | 0.732 |
| Th1/Th2 | 12.18 (8.73, 17.32) | 13.14 (8.16, 22.75) | -0.628 | 0.530 | 0.821 |
| Th17/Treg | 0.38(0.29, 0.50) | 0.35(0.23, 0.49) | 1.340 | 0.180 | 0.821 |
| Th1/Treg | 3.45(2.34, 5.14) | 3.04(1.67 4.50) | 1.726 | 0.084 | 0.554 |
| Th2/Treg | 0.30(0.24, 0.41) | 0.22(0.16, 0.30) | 4.553 | ＜0.001^***^ | ＜0.001^***^ |
| B cell/Treg | 6.82(4.57, 9.81) | 6.36(4.82, 9.58) | 0.298 | 0.766 | 0.936 |
| NK B cell/Treg | 5.60(3.36, 13.24) | 5.10(3.53, 8.13) | 1.222 | 0.222 | 0.821 |

T, T lymphocyte; B, B lymphocyte; NK, natural killer cell, Th1, T-helper 1 cells; Th2, T-helper 2 cells; Th17, T-helper17 cells; Treg, regulatory T cells.

*p < 0.05, **p < 0.01, ***p < 0.001.
