# Supplementary material for: Precision prediction of intervertebral disc degeneration in ankylosing spondylitis using a nomogram model reveals the pivotal role of Th2-type immune dysregulation
Source: Front Immunol. 2025 May 12;16:1556738. doi: 10.3389/fimmu.2025.1556738 (PMC12104166; doi:10.3389/fimmu.2025.1556738)
Supplement: Supplementary file 2 [file Table2.doc]

TABLE S2 Cytokine level in the peripheral blood in the study participants.

|  | **AS+IVDD (n = 60)** | **AS (n = 84)** | **t / z / χ^2^** | **unadjusted P-value** | **BH-adjusted**  **P value** |
| --- | --- | --- | --- | --- | --- |
| IL-2 | 2.04 (1.25, 3.01) | 1.75(1.13, 3.16) | 0.529 | 0.597 | 0.821 |
| IL-4 | 2.24(1.75, 3.56) | 1.87(1.23, 2.72) | 2.648 | 0.008^**^ | 0.088 |
| IL-6 | 4.50(3.13, 6.78) | 5.09(3.33, 7.80) | -0.719 | 0.472 | 0.821 |
| IL-10 | 3.77(2.83, 5.38) | 4.41(3.72, 6.04) | -1.813 | 0.070 | 0.513 |
| IL-17 | 3.70(2.31 9.84) | 3.10(2.41, 4.43) | 1.141 | 0.254 | 0.821 |
| IFN-γ | 3.05(2.20, 4.12) | 2.89(1.81, 3.79) | 1.264 | 0.206 | 0.821 |
| TNF-α | 2.18(1.41, 3.63) | 2.96(1.84, 3.74) | -1.473 | 0.141 | 0.776 |

L-2, interleukin-2; IL-4, interleukin-4; IL-6, interleukin-6; IL-10, interleukin-10; IL-17, interleukin-17; INF-g, interferon-g; TNF-a, tumor necrosis factor-a.

*p < 0.05, **p < 0.01, ***p < 0.001.
