# Supplementary material for: Precision prediction of intervertebral disc degeneration in ankylosing spondylitis using a nomogram model reveals the pivotal role of Th2-type immune dysregulation
Source: Front Immunol. 2025 May 12;16:1556738. doi: 10.3389/fimmu.2025.1556738 (PMC12104166; doi:10.3389/fimmu.2025.1556738)
Supplement: Supplementary file 3 [file Table3.doc]

Table S3 Univariate and multivariate logistic regression analyses were performed for the factors associated with IVDD in patients with AS.

| **Characteristics** | **OR ( 95%CI )** | **univariate logistic regression P-value** | **BH-adjusted P-value** | **Wald χ²** | **Multivariate logistic regression P-value** | **BH-adjusted P-value** | **Wald χ²** |
| --- | --- | --- | --- | --- | --- | --- | --- |
| Gender | 1.025(0.443~2.43) | 0.955 | 0.971 | 0.003 |  |  |  |
| Age | 1.007(0.971~1.045) | 0.718 | 0.862 | 0.139 |  |  |  |
| BMI | 1.363(1.197~1.593) | ＜0.001^***^ | ＜0.001^***^ | 18.042 | ＜0.001^***^ | 0.001^**^ | 12.106 |
| Course of disease | 1.009(0.985~1.034) | 0.469 | 0.862 | 0.523 |  |  |  |
| Smoke | 1.249(0.415~3.629) | 0.684 | 0.862 | 0.162 |  |  |  |
| Drink | 0.923(0.294~2.693) | 0.886 | 0.959 | 0.020 |  |  |  |
| Hypertensi | 1.676(0.571~4.924) | 0.340 | 0.862 | 0.883 |  |  |  |
| Diabetes | 1.5(0.548~4.068) | 0.423 | 0.862 | 0.629 |  |  |  |
| NASIDs | 1.181(0.557~2.52) | 0.665 | 0.862 | 0.187 |  |  |  |
| CS | 0.675(0.238~1.764) | 0.436 | 0.862 | 0.592 |  |  |  |
| CsDMARDs | 1.532(0.583~4) | 0.380 | 0.862 | 0.754 |  |  |  |
| bDMARDs | 0.767(0.103~4.11) | 0.766 | 0.874 | 0.080 |  |  |  |
| WBC | 0.959(0.806~1.127) | 0.617 | 0.862 | 0.240 |  |  |  |
| RBC | 0.874(0.396~1.07) | 0.654 | 0.862 | 0.282 |  |  |  |
| HB | 1.009(0.99~1.031) | 0.356 | 0.862 | 0.749 |  |  |  |
| PLT | 0.995(0.99~1.001) | 0.105 | 0.770 | 3.162 |  |  |  |
| NE | 0.943(0.765~1.144) | 0.559 | 0.862 | 0.327 |  |  |  |
| LYM | 0.887(0.474~1.587) | 0.692 | 0.862 | 0.151 |  |  |  |
| MONO | 4.281(0.988~21.747) | 0.061 | 0.671 | 3.400 |  |  |  |
| ALT | 0.998(0.982~1.004) | 0.585 | 0.862 | 0.125 |  |  |  |
| AST | 0.991(0.946~1.028) | 0.641 | 0.862 | 0.182 |  |  |  |
| TBIL | 0.974(0.888~1.063) | 0.559 | 0.862 | 0.330 |  |  |  |
| DBIL | 1.048(0.799~1.374) | 0.716 | 0.862 | 0.115 |  |  |  |
| IBIL | 1.016(0.972~1.082) | 0.481 | 0.862 | 0.337 |  |  |  |
| Cr | 0.99(0.966~1.012) | 0.416 | 0.862 | 0.717 |  |  |  |
| CHOL | 0.981(0.664~1.441) | 0.922 | 0.971 | 0.009 |  |  |  |
| TG | 0.917(0.599~1.344) | 0.667 | 0.862 | 0.177 |  |  |  |
| HDL | 1.122(0.382~3.262) | 0.831 | 0.930 | 0.044 |  |  |  |
| LDL | 0.654(0.386~0.996) | 0.095 | 0.770 | 3.084 |  |  |  |
| ESR | 1.005(0.985~1.025) | 0.615 | 0.862 | 0.241 |  |  |  |
| CRP | 1.028(1.004~1.06) | 0.040^*^ | 0.528 | 3.978 |  |  |  |
| IgA | 1.217(0.995~1.545) | 0.073 | 0.688 | 3.061 |  |  |  |
| IgG | 0.953(0.852~1.059) | 0.379 | 0.862 | 0.753 |  |  |  |
| IgM | 0.978(0.812~1.05) | 0.626 | 0.862 | 0.115 |  |  |  |
| Total T | 1(0.999~1.001) | 0.570 | 0.862 | 0.000 |  |  |  |
| T% | 0.988(0.946~1.032) | 0.587 | 0.862 | 0.296 |  |  |  |
| Total B | 0.999(0.996~1.002) | 0.685 | 0.862 | 0.426 |  |  |  |
| B% | 0.957(0.887~1.029) | 0.240 | 0.862 | 1.346 |  |  |  |
| CD4^+^T | 1(0.998~1.001) | 0.509 | 0.862 | 0.000 |  |  |  |
| CD4^+^T% | 0.97(0.93~1.02) | 0.277 | 0.862 | 1.671 |  |  |  |
| CD8^+^T | 1(0.998~1.002) | 0.958 | 0.971 | 0.000 |  |  |  |
| CD8^+^T% | 1.015(0.969~1.065) | 0.528 | 0.862 | 0.382 |  |  |  |
| CD4^+^T/CD8^+^T | 0.752(0.45~0.999) | 0.257 | 0.862 | 1.963 |  |  |  |
| NK | 1.002(0.999~1.005) | 0.208 | 0.862 | 1.711 |  |  |  |
| NK% | 1.024(0.972~1.081) | 0.372 | 0.862 | 0.765 |  |  |  |
| Th1% | 1.004(0.958~1.051) | 0.869 | 0.956 | 0.029 |  |  |  |
| Th2% | 6.769(2.69~19.491) | ＜0.001^***^ | ＜0.001^***^ | 14.328 | 0.015^*^ | 0.019^*^ | 5.921 |
| Th17% | 1.418(0.873~2.379) | 0.166 | 0.862 | 1.865 |  |  |  |
| Treg% | 0.922(0.683~1.236) | 0.587 | 0.862 | 0.288 |  |  |  |
| Th1 | 0.998(0.993~1.003) | 0.514 | 0.862 | 0.613 |  |  |  |
| Th2 | 1.136(1.044~1.249) | 0.005^**^ | 0.099 | 7.773 | 0.838 | 0.838 | 0.042 |
| Th17 | 1.012(0.955~1.071) | 0.687 | 0.862 | 0.166 |  |  |  |
| Treg | 0.99(0.964~1.015) | 0.426 | 0.862 | 0.584 |  |  |  |
| Th1/Th2 | 0.973(0.931~1.012) | 0.188 | 0.862 | 1.654 |  |  |  |
| Th17/Treg | 2.32(0.448~13.336) | 0.318 | 0.862 | 0.945 |  |  |  |
| Th1/Treg | 1.023(0.881~1.185) | 0.760 | 0.874 | 0.090 |  |  |  |
| Th2/Treg | 1.019(0.527~1.794) | 0.937 | 0.971 | 0.004 |  |  |  |
| B cell/Treg | 1.023(0.939~1.115) | 0.596 | 0.862 | 0.269 |  |  |  |
| NK B cell/Treg | 1.03(0.981~1.089) | 0.254 | 0.862 | 1.231 |  |  |  |
| IL-2 | 1.049(0.82~1.34) | 0.697 | 0.862 | 0.146 |  |  |  |
| IL-4 | 1.407(1.128~1.843) | 0.006^**^ | 0.099 | 7.433 | 0.012^*^ | 0.019^*^ | 6.343 |
| IL-6 | 1.007(0.973~1.043) | 0.668 | 0.862 | 0.155 |  |  |  |
| IL-10 | 0.895(0.734~1.07) | 0.242 | 0.862 | 1.331 |  |  |  |
| IL-17 | 1.006(0.963~1.05) | 0.768 | 0.874 | 0.074 |  |  |  |
| IFN-γ | 1.014(0.974~1.065) | 0.496 | 0.862 | 0.372 |  |  |  |
| TNF-α | 0.997(0.822~1.193) | 0.971 | 0.971 | 0.001 |  |  |  |

OR, odds ratio; 95%CI:95% confidence interval;

*p<0.05,**p<0.01,***p<0.001.
