# Supplementary figures and images for: Precision prediction of intervertebral disc degeneration in ankylosing spondylitis using a nomogram model reveals the pivotal role of Th2-type immune dysregulation
Source: Front Immunol. 2025 May 12;16:1556738. doi: 10.3389/fimmu.2025.1556738 (PMC12104166; doi:10.3389/fimmu.2025.1556738)

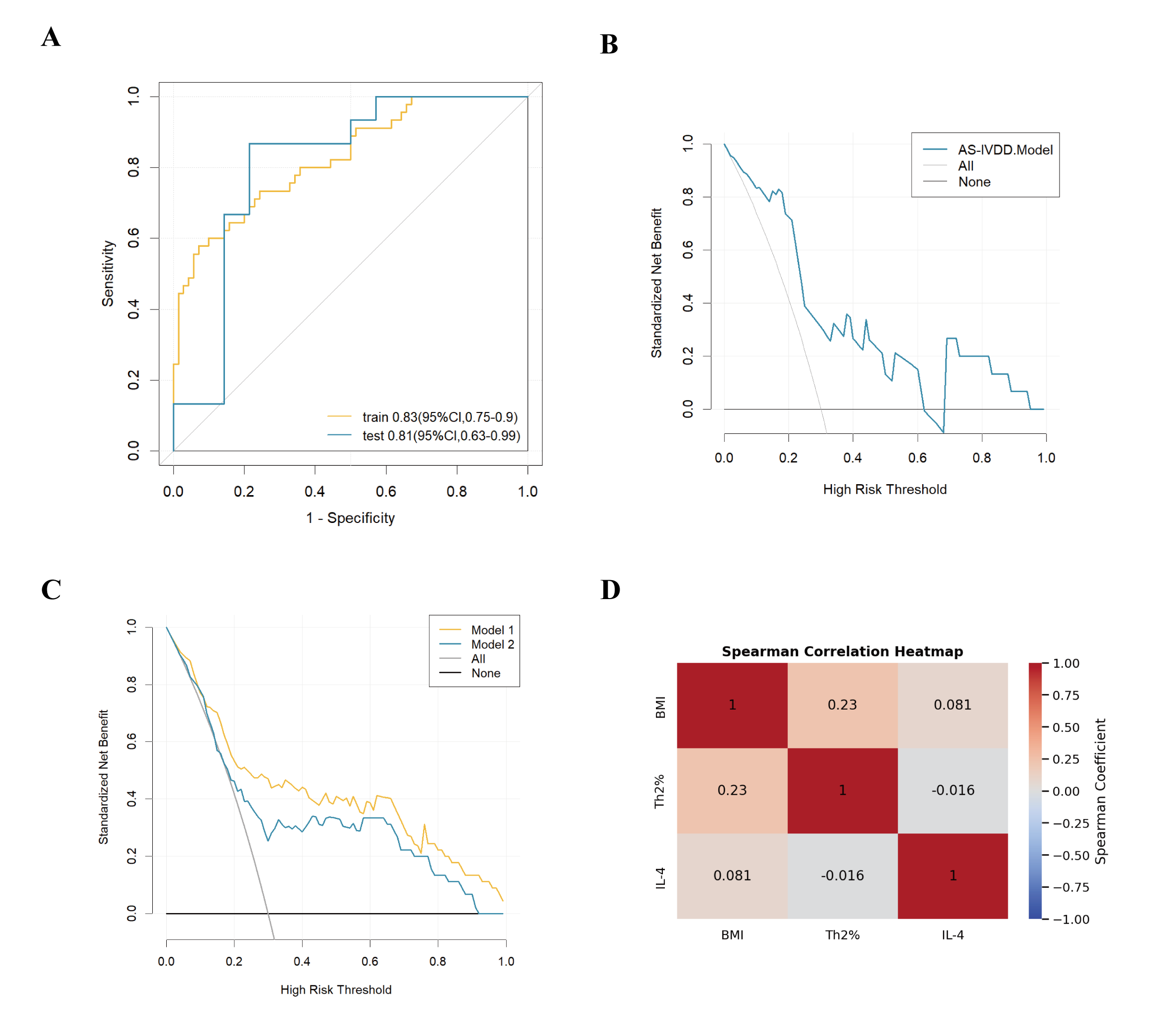

Supplement: Supplementary Figure 1 — (A) The ROC curve for the discrimination of the nomogram to predict the risk of IVDD in training and validation cohorts. (B) DCA for predicting the risk of IVDD in AS patients in validation cohorts. (C) DCA demonstrated that Model 1 consistently provided greater net clinical benefit than Model 2 across a threshold probability range of 10% to 100%, supporting its superior utility in guiding clinical decision-making. (D) Spearman correlation analysis was performed to assess the relationships between BMI and immune parameters IL-4 and Th2%. The color gradient represents the strength and direction of correlation (ρ), with red indicating positive and blue indicating negative associations. A weak negative correlation was observed between BMI and IL-4 (ρ = -0.13, p = 0.22), while a weak positive correlation was found between BMI and Th2% (ρ = 0.12, p = 0.27). However, neither reached statistical significance, suggesting no meaningful correlation between BMI and these immune markers in the current dataset. [file Image1.tif]
